# Influence of Hydrocarbon-Oxidizing Bacteria on the Growth, Biochemical Characteristics, and Hormonal Status of Barley Plants and the Content of Petroleum Hydrocarbons in the Soil

**DOI:** 10.3390/plants10081745

**Published:** 2021-08-23

**Authors:** Elena Kuzina, Gulnaz Rafikova, Lidiya Vysotskaya, Tatyana Arkhipova, Margarita Bakaeva, Dar’ya Chetverikova, Guzel Kudoyarova, Tatyana Korshunova, Sergey Chetverikov

**Affiliations:** Ufa Institute of Biology, Ufa Federal Research Centre, Russian Academy of Sciences, 450054 Ufa, Russia; misshalen@mail.ru (E.K.); rgf07@mail.ru (G.R.); vysotskaya@anrb.ru (L.V.); tnarkhipova@mail.ru (T.A.); margo22@yandex.ru (M.B.); belka-strelka8031@yandex.ru (D.C.); guzel@anrb.ru (G.K.); che-kov@mail.ru (S.C.)

**Keywords:** petroleum contamination, *Enterobacter*, *Pseudomonas*, *Hordeum vulgare* L., plant hormones, chlorophyll, flavonoids, nitrogen balance index, proline

## Abstract

Much attention is paid to the relationship between bacteria and plants in the process of the bioremediation of oil-contaminated soils, but the effect of petroleum degrading bacteria that synthesize phytohormones on the content and distribution of these compounds in plants is poorly studied. The goal of the present field experiment was to study the effects of hydrocarbon-oxidizing bacteria that produce auxins on the growth, biochemical characteristics, and hormonal status of barley plants in the presence of oil, as well as assessing the effect of bacteria and plants separately and in association with the content of oil hydrocarbons in the soil. The treatment of plants with strains of *Enterobacter* sp. UOM 3 and *Pseudomonas hunanensis* IB C7 led to an increase in the length and mass of roots and shoots and the leaf surface index, and an improvement in some parameters of the elements of the crop structure, which were suppressed by the pollutant. The most noticeable effect of bacteria on the plant hormonal system was a decrease in the accumulation of abscisic acid. The data obtained indicate that the introduction of microorganisms weakened the negative effects on plants under abiotic stress caused by the presence of oil. Plant-bacteria associations were more effective in reducing the content of hydrocarbons in the soil and increasing its microbiological activity than when either organism was used individually.

## 1. Introduction

The industrial process of petroleum extraction and refinery leads to the global pollution of ecosystems with hydrocarbons. In comparison with water and air, the soil environment is the most susceptible to the negative impact of these pollutants, which causes a decrease in the biological activity of the soil and a loss of its main quality, i.e., fertility [1,2,3]. The most environmentally friendly and economically feasible solution to this problem is the use of biological technologies and, in particular, microbial-plant associations. They consist of microorganisms that destroy organic pollutants or transform them into less toxic compounds and of plants that create optimal conditions for the existence and reproduction of bacteria [4]. Roots provide a surface for the attachment of microorganisms and secrete exudates contributing to an increase in their number in the rhizosphere [5,6], and synthesize enzymes that degrade organic substrates in the soil [7]. In general, root development increases the porosity of the soil, which enhances the mass transfer of substrate and electron acceptors during the oxidation of oil components [8]. Rhizosphere microorganisms, in turn, intensify plant growth by releasing various biologically active substances and improve phosphorus and nitrogen nutrition [9,10]. They increase stress resistance by activating the antioxidant system in plants [11] and protect them against infection due to antagonistic interactions between microorganisms and pathogenic agents. The listed bacteria-induced mechanisms help plants to cope with the adverse conditions of oil pollution. Thus, the interaction of plants and microorganisms in oil-contaminated soil seems to be an ideal example of a mutually beneficial partnership that can be used in the processes of cleaning and restoration of anthropogenically disturbed territories. However, despite the active study of bacterial effects on plants during the process of the bioremediation of soils contaminated with hydrocarbons, insufficient attention has been paid to some of its aspects. For example, the effects of oil-degrading bacteria capable of synthesizing plant hormones on the content and distribution of hormones in plants have not been studied, although in the case of some other stress factors (drought, salinity) such experiments have been carried out [12,13]. To fill this gap, we carried out a number of laboratory experiments [14,15]. The results obtained from them were used in the design of the field experiment. The need for these experiments was dictated by the fact that the Russian Federation possesses a significant oil and gas complex and, therefore, the problem of cleaning soils contaminated with hydrocarbons is very important for this country. The purpose of this study was to deepen knowledge on the effects of hydrocarbon-oxidizing auxin-producing bacteria on the growth, biochemical parameters, and hormonal status of barley plants in the presence of oil. Furthermore, the work included the assessment of the effectiveness of bacteria and plants separately and in association with reducing the content of hydrocarbons in the soil, which is important for the development of environmentally friendly approaches to cleaning and restoring anthropogenically disturbed soils. We assumed that the ability of the hydrocarbon-oxidizing auxin-producing bacteria *Enterobacter* sp. UOM 3 and *Pseudomonas hunanensis* IB C7 to stimulate the growth and development of barley plants against the background of oil pollution and reduce the content of oil hydrocarbons in the soil, will remain in field conditions.

## 2. Results

### 2.1. The Growth of Plants under the Influence of Oil and Bacteria

**Figure 1:**
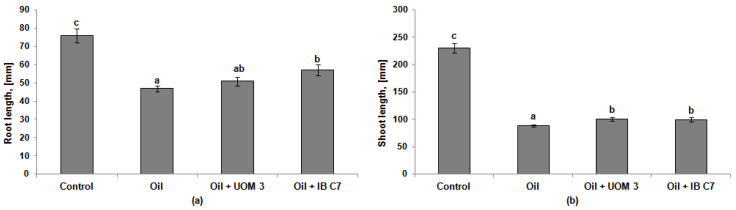
Root (**a**) and shoot (**b**) length of barley plants 10 days after germination. UOM 3 and IB C7, variants of experiments with introduction of *Enterobacter* sp. UOM 3 and *P.* *hunanensis* IB C7, respectively. Statistically different means values for each indicator (n = 15) are marked with different letters (*p* ≤ 0.05).

The presence of oil inhibited the growth of roots and shoots at the initial stage of plant development. The length of these organs was 1.6 and 2.6 times less than in the control, respectively (Figure 1). When treated with the *P. hunanensis* IB C7 strain, the length of the roots increased in comparison with the plants untreated with bacteria and grown in oil-contaminated soil. Inoculation with *Enterobacter* sp. UOM 3 and *P. hunanensis* IB C7 resulted in a 12–13% increase in shoot elongation. A similar trend was noted when analyzing the fresh weight of roots and shoots (Table 1). Under the influence of oil, a 27% and 80% decline in the fresh weight of roots and shoots, respectively, was detected, while the ratio of the root-to-shoot mass increased from 0.11 in the control to 0.37–0.49 in the contaminated soil. When using the bacterial strain *P. hunanensis* IB C7, a significant increase in the mass of roots and shoots was observed in comparison with these indicators in oil-contaminated soil without treatment.

**Figure 2:**
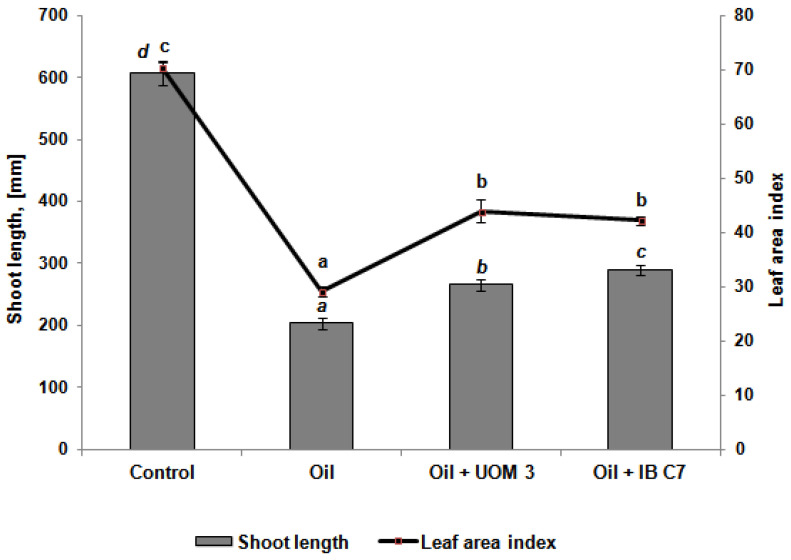
Shoot length and leaf area index of barley plants 34 days after germination. UOM 3 and IB C7, variants of experiments with introduction of *Enterobacter* sp. UOM 3 and *p.* *hunanensis* IB C7, respectively. Statistically different means values for each indicator (*n* = 50) are marked with different letters (*p* ≤ 0.05).

The determination of the height of the aboveground part of the plants 34 days after the emergence of shoots showed, in general, the preservation of the regularities of changes in the growth of shoots (Figure 2). The inhibitory effect of oil on the growth of barley plants did not decrease over time: the height of plants grown in contaminated soil was three times lower than in clean soil. Bacterization had a beneficial effect on plants: when treated with strains of *Enterobacter* sp. UOM 3 and *P. hunanensis* IB C7, their heights were 31% and 43% higher, respectively, than in unbacterized plants grown in the oil-contaminated soil. The leaf surface index of plants exposed to oil was 2.4 times lower than in the control (Figure 2). The introduction of *Enterobacter* sp. UOM 3 and *P. hunanensis* IB C7 increased the leaf surface index by 45% and 50%, respectively. 

The evaluation of the effects of oil and bacterization on some indicators of the growth and development of barley plants at the end of the experiment is presented in Table 2. Soil pollution resulted in a 2.8-fold decline in bushiness, a 2.6-fold decline in shoot mass, and a 1.9-fold decline in shoot height. The treatment of plants with bacterial strains had no promotive effect on the first indicator but increased the second indicator by 15%. In addition, the *Enterobacter* sp. UOM 3 contributed to shoot elongation by 5%.

The number of ears formed in plants grown in contaminated soil was 2.2 times less than in pure soil. It increased by 22% with the introduction of the *P. hunanensis* IB C7 strain when compared with this indicator in untreated plants in soil with oil. The pollutant had the strongest inhibitory effect on a spike: against the background of oil pollution, the length of the main spike decreased by 3.5 times, and the number of spikelets per spike decreased by 2.9 times. The use of bacteria led to an increase in these parameters. A more pronounced positive effect was exerted by the *Enterobacter* sp. UOM 3, whose treatment increased the length of the main spike and the number of spikelets in the spike by 37% and 12%, respectively.

### 2.2. The Number of Microorganisms and the Content of Hydrocarbons in the Soil

**Figure 3:**
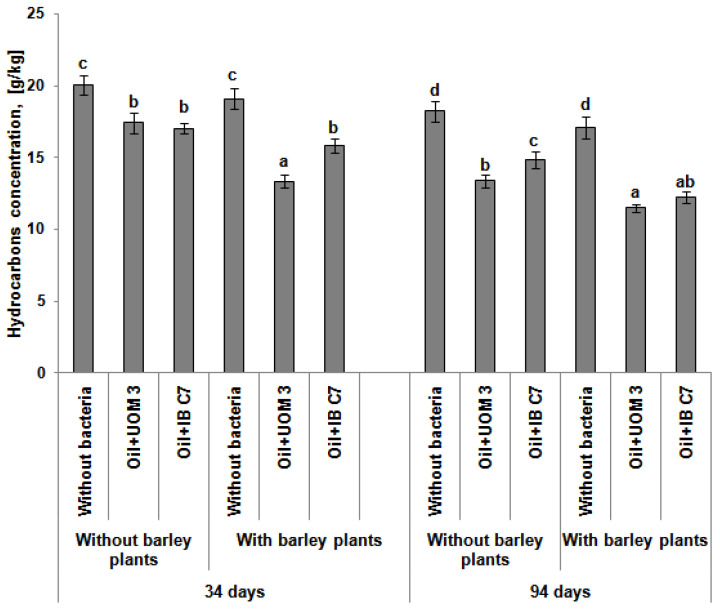
Hydrocarbon content in the soil 34 and 94 days after germination. UOM 3 and IB, variants of experiments with introduction of *Enterobacter* sp. UOM 3 and *P.* *hunanensis* IB C7, respectively. Statistically different means values for each indicator are marked with different letters (*p* ≤ 0.05).

The introduction of hydrocarbon-oxidizing bacteria accelerated the process of oil decomposition. Thus, by the end of the experiment, the introduction of *Enterobacter* sp. UOM 3 and *P. hunanensis* IB C7 reduced the content of hydrocarbons by 26% and 18%, respectively, compared to the variant without the introduction of bacteria (Figure 3). The combined use of hydrocarbon-oxidizing microorganisms and plants was from 29% to 33% more effective than the option where the plants were not subjected to bacterial treatment. 

The number of heterotrophic microorganisms in the contaminated soil in the absence of plants remained at the same level throughout the experiment (Table 3). The addition of oil-degrading bacteria increased this indicator by the end of the experiment. In the soil with plants, the total number of microorganisms was higher than in the soil without plants. The introduction of *Enterobacter* sp. UOM 3 and *P. hunanensis* IB C7 into contaminated soil with plants increased the number of heterotrophic microorganisms between 1.6 and 2.2 times.

One of the most important criteria indicating the success of oil biodegradation is the survival rate of the introduced hydrocarbon-oxidizing bacteria, i.e., their ability to maintain high numbers for a long time. As in the case of heterotrophic microorganisms, the density of the population of hydrocarbon-oxidizing bacteria in the soil with oil without plants remained stable throughout the experiment. The addition of *Enterobacter* sp. UOM 3 increased the number of hydrocarbon-oxidizing bacteria by an order of magnitude by the end of the experiment. In the experiments with plants, it was slightly higher than in the soil without plants. In general, the degree of destruction of hydrocarbons correlated with the amount of *Enterobacter* sp. UOM 3 (r = 0.45, *p* ≤ 0.05).

The number of oligonitrophilic microorganisms in contaminated soil without plants and bacterization slightly increased over time. When both strains were used in the experiments without plants, the number of microorganisms in this group increased between 4.6 and 5.3 times. In the soil with barley plants, this indicator was higher than in the soil with the absence of plants. By the end of the experiment, the number of oligonitrophils increased between 2.4 and 3.5 times with the introduction of oil-degrading bacteria into the soil with plants compared to the experiments with plants but without bacterial treatment.

### 2.3. The Effect of Oil and Bacteria on the Content of Hormones in Plants

**Figure 4:**
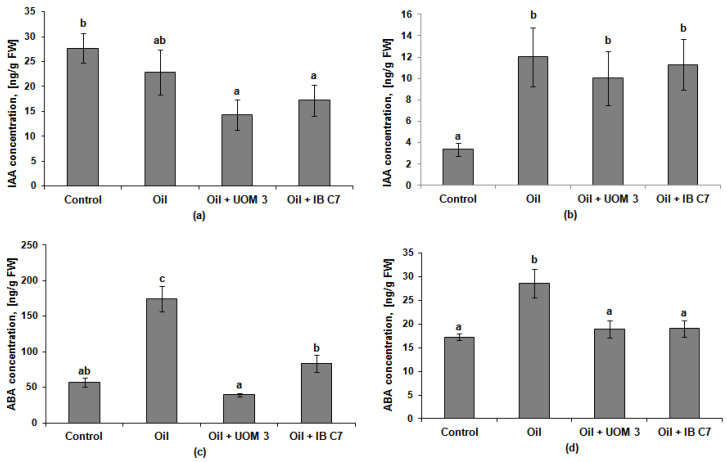
Indole-3-acetic acid (IAA) and abscisic acid (ABA) content in roots ((**a**,**c**), respectively) and shoots ((**b**,**d**), respectively) of barley plants. UOM 3 and IB C7, variants of experiments with introduction of *Enterobacter* sp. UOM 3 and *P.* *hunanensis* IB C7, respectively. Statistically different means values for each indicator (*n* = 9) are marked with different letters (*p* ≤ 0.05).

No significant differences were found in the content of auxin, indole-3-acetic acid (IAA), in barley roots either without oil or in its presence (Figure 4). In the experiments where seeds were treated with *Enterobacter* sp. UOM 3 and *P. hunanensis* IB C7, IAA content in plant roots was 1.6 and 1.9 times lower than in the control, respectively. In the experiments without oil, IAA was significantly higher in the roots than in the barley shoots. In the presence of the pollutant, the level of this hormone in the aboveground and underground parts of the plants leveled off. Compared to the control, the IAA content in the shoots of barley grown in soil with the pollutant increased approximately 3 times.

The content of abscisic acid in the shoots in all variants of the experiment was lower than in the roots (on average, from 2 to 6 times). Against the background of oil pollution, an increase in the abscisic acid (ABA) level was found only in non-inoculated plants (Figure 4). This was more noticeable in the roots, where its concentration increased 3.1 times. The use of microorganisms for treating plants planted in soil with oil led to the decline in ABA content to the control level both in roots and shoots.

**Figure 5:**
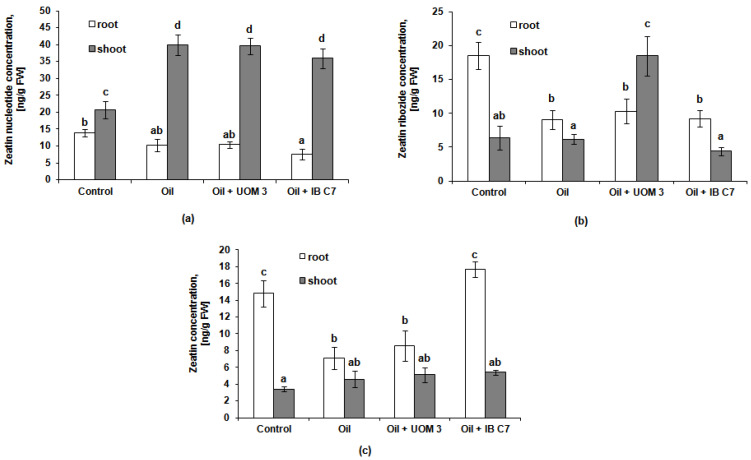
Zeatin nucleotide (**a**), zeatin ribozide (**b**), zeatin (**c**) content in roots and shoots of barley plants. UOM 3 and IB C7, variants of experiments with introduction of *Enterobacter* sp. UOM 3 and *P.* *hunanensis* IB C7, respectively. Statistically different means values for each indicator (n = 9) are marked with different letters (*p* ≤ 0.05).

The content of all three analyzed forms of cytokinin in the roots of barley plants was approximately 14–19 ng/g fresh weight in the control (Figure 5). Under the influence of oil, the content of zeatin and its ribolized form decreased most noticeably (2 times in each case). The introduction of bacterial strains against the background of contamination did not affect the level of zeatin nucleotide and zeatin riboside in the plant roots; it remained practically the same as in unbacterized plants grown in the presence of oil. At the same time, barley plants growing in the presence of oil responded to the treatment with the *P. hunanensis* IB C7 strain by an increase in the free form of zeatin in the roots. The content of zeatin and zeatin riboside in the shoots was lower than in the roots in all variants of the experiment (Figure 5), with the exception of a sharp (almost threefold) increase in the amount of zeatin riboside when using the *Enterobacter* sp. UOM 3 for the bacterization of plants in contaminated soil. Oil pollution was the impetus for the accumulation of zeatin nucleotide in the shoots: in the plants planted in the soil with oil, it was between 1.7 and 1.9 times more than in the control (plants grown without both oil and bacterization).

### 2.4. The Effect of Oil and Bacteria on the Synthesis of Plant Pigments and Proline

Ten days after the emergence of seedlings, the content of chlorophyll in barley shoots measured in plants growing in the presence of oil was 2 times lower than in the control (Table 4). The same trend persisted afterward. In cases when plants in contaminated soil were treated with bacterial strains, it was found to be between 22.2% and 33.3% more than in the version with oil but without bacterization.

The minimum level of flavonoids was found in the control plants (Table 4). The presence of oil in the soil led to a 1.6-fold increase in the amounts of flavonoids in young plants, and with the repeated sampling of older plants, it was 1.1 times higher under stress compared to the control. At the beginning of the growing season, inoculation with bacteria did not affect the accumulation of these pigments. With the further development of plants, the content of flavonoids in unbacterized barley growing in soil with oil became 7%–10% lower than in bacterized plants. 

Changes in chlorophyll and flavonoid content in plants over time, as well as under the influence of oil pollution, are clearly described by the plant nitrogen balance index (NBI), which is an indicator of changes in the C/N ratio in leaves. In the control plants, it was in the range of between 49.2 and 62.7 conventional units during the measurement period; ten days after the emergence of seedlings under stress conditions, it decreased to between 18.1 and 20.9, and later increased to between 24.7 and 30.5. The highest NBI value was reached in the variant with the inoculation of plants with *P. hunanensis* IB C7 (Table 4).

At the initial stage of the growing season, the content of proline in the barley shoots of the control plants was 26.4 μg/g fresh weight (Table 4). In the presence of oil, its amount increased 3 times (79.3 μg/g). However, in the variants where the bacterial strains were introduced against the background of the pollutant, the amount of this amino acid in the leaves was noticeably lower. In plants treated with *P. hunanensis* IB C7, the proline content was 35.5 μg/g, while the use of *Enterobacter* sp. UOM 3 decreased its level down to the control value. In the course of the experiment, the proline level increased in all variants of the experiment by an average of between 1.5 and 2.3 times. At the same time, in cases where bacterial treatment was carried out against the background of oil pollution, its amount, as before, was significantly lower than in the variant with oil without the introduction of microorganisms (by between 30% and 47%).

## 3. Discussion

The process of seed germination in oil-contaminated soil is known to differ significantly in different plant species [16,17]. In the present study, as in the work of Ali [18], there was no negative effect of hydrocarbons on the germination of barley plants, which was 89%, and did not differ significantly from that in the control plants (91%). The high germination capacity of seeds under conditions of oil pollution may be due to the fact that, during germination, the seeds use the reserve nutrients they contain. Later on, as the plants develop and the supply of nutrients is depleted, unfavorable conditions in the soil associated with the presence of oil (deficiency of moisture, nutrients, air, etc.) inhibit their growth. There is an opinion [19,20], that the main indicator of the detrimental effect of petroleum hydrocarbons on plants is growth retardation leading to a decrease in biomass. Throughout the experiment, oil exerted an inhibitory effect on all analyzed parameters of the growth and development of barley plants, which could be explained by its direct toxic effect [21,22]. On the other hand, the soil contamination with petroleum leads to a decrease in its water-holding capacity and aeration, as well as to a change in a number of chemical properties (e.g., pH), and the availability of mineral nutrients and enzyme activity [3,23]. All of these factors, taken together, could lead to the inhibition of the growth and development of barley plants in contaminated soil (Table 1 and Table 2, and Figure 1 and Figure 2). Such a response to petroleum contamination is characteristic for plants of the *Poaceae* family [18,24]. 

The introduction of microorganisms partially compensated for the adverse effect of the pollutant. The positive effect of bacterization was likely due to both the accelerated degradation of the pollutant and the bacterial production of substances promoting the growth and development of plants [10,25].

The capacity of bacteria to produce hormones is considered one of the most important mechanisms of their effect on plant growth and development [26,27,28]. The strains used in this study degrade oil and petroleum products and produce IAA [14,29]. Microbial synthesis of the phytohormone auxin has been known for a long time [26]. The capacity of *Enterobacter* sp. UOM 3 and *P. hunanensis* IB C7 to increase the length and mass of shoots and roots detected in laboratory experiments against the background of hydrocarbon stress [14,15] was also manifested in the present field experiments. 

Plant inoculation with growth-stimulating bacteria has been shown to increase plant growth and resistance to stressful influences such as toxic metals [30], salinity [12,31], and petroleum hydrocarbons [32]. These effects are attributed to microbial production and the provision of auxins and other hormones to plants [28,33,34,35]. Hormones are known to act on plant growth and development not individually but through a cross-talk of interrelated signals. At the same time, it is still not fully understood how the mutual influence of these biologically active substances helps plants to cope with stress. Information about the capacity of bacteria to produce plant hormones is not sufficient for understanding the mechanism of their growth-promoting action and it is important to follow the bacterial effects on hormonal content *in planta*. The importance of bacterial effects on the hormone content in plants was demonstrated under salinity [13] and soil pollution with toxic metals [36]. However, less attention was paid to the hormonal status of plants growing in oil-contaminated soil. Reports on this theme are scarce and corresponding experiments have been performed in laboratories [15,37]. In the present field experiment, the effect of oil pollution on the hormonal system of barley plants was manifested in an increase in the IAA level in the shoots and a decrease in its content in the roots (Figure 4a,b). Such a change in the distribution of hormones may be the result of the inhibition of their transport along the phloem from the shoots to the roots. It was previously described that the accumulation of auxins in plant shoots and the inhibition of their outflow to the roots occurs under the influence of flavonoids [38,39]. Consequently, the increase in the content of these pigments in the presence of oil (Table 4) may be related to the regulation of auxin distribution in barley plants. The accumulation of IAA in shoots could protect plants from the oxidative stress-accompanying action of many stress factors [40] since this hormone is known to be capable of activating the antioxidant system [8]. The absence of the effect of bacteria capable of producing auxins in vitro [14,29] on the IAA content in barley plants was unexpected (Figure 4a,b). It is possible that an increase in its concentration under the influence of microorganisms was not observed due to the high level of flavonoids activating oxidative degradation of auxins [38].

The presence of oil in the soil inhibited root growth to a lesser extent than shoot growth. The redistribution of biomass in favor of roots is a characteristic growth response to a deficiency of water and mineral nutrients [41,42]. Since the presence of a pollutant reduces the availability of water and ions to plants, maintaining root growth is an important plant response to adapt to these stressful conditions. On the other hand, root development is essential for the bacterial colonization of the rhizosphere. Cytokinins are able to promote shoot growth but inhibit root growth [43]. In the present experiment, under the influence of pollution, a relative (compared to shoot) activation of root growth was observed manifesting itself in an increased root-to-shoot mass ratio (Table 1) accompanied by a decrease in the content of cytokinins in the roots (Figure 5). In this case, a decrease in the level of these hormones in underground organs can be considered as one of the mechanisms enabling the activation of root growth. In the presence of oil, a decrease in the root zeatin riboside, which is a transport form of cytokinins, may indicate a redistribution of cytokinins from roots to shoots. The increased content of cytokinins in plant shoots under the influence of pollution was accompanied by the activation of their growth only upon the introduction of *Enterobacter* sp. UOM 3 (Figure 5). Shoot growth promotion under the influence of an increased level of cytokinins could be prevented by the accumulation of ABA, whose content, in the absence of bacterization, increased in the shoots by 1.7 times in comparison with plants that grew in clean soil. ABA is known to be an antagonist of cytokinins in the regulation of plant growth [44]. ABA content decreased under the influence of *Enterobacter* sp. UOM 3 and *P. hunanensis* IB C7 down to the control values, leading to an increase in the ratio of the total amount of cytokinins to the amount of ABA by 1.4 and 1.9 times, respectively, which may explain the detected trend towards the promotion of shoot growth under the influence of bacterization. The decreased accumulation of ABA in plants was the most noticeable bacterial effect on the plant hormonal system. The accumulation of this hormone is an indicator of unfavorable conditions for plant growth (first of all, a deficiency of water and mineral nutrients) [45]. Bacterization reduced the content of hydrocarbons in the soil, which could improve the supply of plants with water and mineral nutrients resulting in a reduced ABA level in bacterized plants.

The inhibition of shoot growth under conditions of water shortage resulting from oil pollution leads to the formation of smaller leaves, which is reflected in a decrease in the leaf surface index by 2.4 times compared with control plants grown in clean soil (Figure 2). This is consistent with the results of our earlier research [15]. Inoculation with bacterial strains led to a decrease in the ABA content (Figure 4c,d), which could contribute to an increase in stomatal conductance, coupled with an increased rate of photosynthesis, and lead to an increase in the leaf surface index (Figure 2). 

It has been shown that hydrocarbons have an inhibitory effect on photosynthesis [46,47] and the content of chlorophyll, in particular [48,49,50]. It is even proposed that the decrease in the amount of chlorophyll is an indicator of environmental pollution [51]. In the present research, chlorophyll content in barley leaves decreased 2.2 times with the presence of oil (Table 4), while bacterization did not influence this indicator at the initial stages of plant development. However, chlorophyll content increased with time under the bacterial treatment of plants when compared to the control, which serves as evidence of a decline in the level of pollutants in the soil with introduced bacteria (Figure 3). 

Plants use an increase in the synthesis of flavonoids and proline as mechanisms of adaptation to stressful environments [52,53,54,55]. Flavonoids are considered indicators of nitrogen availability in a plant [56], the absorption and assimilation of which plays an important role in plant growth and development [57]. According to the hypothesis of a balance between growth and differentiation [58], the content of flavonoids increases with a low availability of nitrogen and, as a rule, is inversely proportional to the content of chlorophyll [59]. Therefore, the ratio between the amount of chlorophyll and flavonoids, known as the nitrogen balance index (NBI), has been proposed as an indicator of the nitrogen status of plants [59,60,61]. Plants grown in oil-contaminated soil showed the lowest NBI value, significantly different from the values obtained in the control plants (Table 4). Based on the above hypothesis, a decrease in chlorophyll production and a parallel increase in flavonoid content can be interpreted as a result of the low availability of nitrogen as a result of oil pollution. A slight increase in NBI as a result of bacterization by the *P. hunanensis* IB C7 may be due to its nitrogen-fixing ability [14], but this assumption needs further study. 

One of the early adaptive reactions of plants to unfavorable environmental conditions is an increase in the synthesis of various low-molecular compounds, e.g., proline. It participates in the regulation of the osmotic potential of cells, stabilizes the cell structure, and removes excess ROS, thereby increasing the resistance of plants to stress [62]. Data on changes in the level of this amino acid in plants at different concentrations of oil in the soil are quite contradictory and are determined both by the type of pollutant and the type (and even variety) of plants [20]. For example, the presence of diesel fuel and gasoline in soil has been shown to increase the accumulation of this amino acid in wheat plants, while pollution with oil products caused its decrease in the leaves of bean plants [63,64]. In the present experiment, the presence of oil led to a sharp increase in its content in the leaves compared to the control (Table 4). A decrease in the amount of proline resulting from bacterization suggests that the introduction of oil-destroying strains reduces the level of abiotic stress caused by the presence of toxic substances in the soil.

The introduction of hydrocarbon-oxidizing bacteria significantly increased the decomposition of the pollutant (Figure 3), which is probably due to the high survival rate and active functioning of the introduced microbial population. The simultaneous use of plants and bacteria led to an acceleration of the degradation of hydrocarbons in the soil compared to the options without plants. This is due to an increase in the microbial biomass in the rhizosphere of plants (Table 3), whose root system creates a comfortable environment for the growth of microorganisms due to the release of substrate for the growth of microorganisms [5,6]. In addition, root development improves soil aeration by creating air channels, which is important for the aerobic microbiota [65].

A significant contribution to the number of heterotrophic microorganisms in the soil was made by hydrocarbon-oxidizing microorganisms (Table 3). This is confirmed by the same tendency in the change of the number of both ecologo-trophic groups (correlation coefficient r = 0.981, *p* ≤ 0.5). An increase in the pool of oligonitrophilic microorganisms was noted over time, most noticeable in variants with the introduction of strains. Obviously, this was due to a decrease in the toxicity of the soil brought about by a decrease in the oil content in it (both as a result of evaporation and biological decomposition), since this group of microorganisms is sensitive to contamination with various pollutants, including hydrocarbons [66,67].

## 4. Materials and Methods

### 4.1. Plant Growth Conditions and Treatments 

The study was carried out in the territory of the Ufa district of the Republic of Bashkortostan (Russia) from the 2 June to 8 September 2020. Weather indicators during this period were within the average statistical parameters for the previous five years. In our work, we used barley plants (*Hordeum vulgare* L.), variety Chelyabinskiy 99, adapted to the climatic conditions of the Ural region, in which the territory of the Republic of Bashkortostan is located. Barley was chosen, out of seven plant species, as relatively oil-resistant and highly sensitive to inoculation with hydrocarbon-oxidizing bacteria [14,68,69]. We studied the association of barley with petroleum-degrading strains *Enterobacter* sp. UOM 3 and *Pseudomonas hunanensis* IB C7, synthesizing IAA [14,29]. Plant seeds were provided by the Bashkir Research Institute of Agriculture (Ufa, Russian Federation). Bacterial strains *Enterobacter* sp. UOM 3 (isolated by the authors from a sample of urban soil from Jebra Island (Tunisian Republic)) and *P. hunanensis* IB C7 (isolated by the authors from the steppe soil of the Orenburg region, Russian Federation) were stored in the collection of microorganisms of the Ufa Institute of Biology, RAS (Ufa, Russian Federation) [14,29]. The experimental site, whose soil (clay-illuvial chernozem, 3.7% organic carbon, 6.6% humus, pH of the aqueous extract of 5.7) was contaminated with commercial oil of the Urals brand, was divided into 1.5 m^2^ plots. Commercial oil of the Urals brand is heavy (density 860–871 kg/m^3^), sulfurous (sulfur content 1.2–1.3%) oil, which is a mixture of light West Siberian oil and heavy high-sulfur oil from the Ural and Volga oil fields. The contamination was caused by a small oil leak from the oil pipeline. Samples for chemical analysis were taken from the top layer (0–20 cm) of the soil two months after the oil spill. The average content of the pollutant was 27 g/kg of soil. The experiment was carried out in seven variants with three replications of each:

1. Clean soil + barley plants without bacterial treatment (control);
2. Oil-contaminated soil;
3. Oil-contaminated soil + barley plants without bacterial treatment;
4. Oil-contaminated soil + *Enterobacter* sp. UOM 3;
5. Oil-contaminated soil + *Enterobacter* sp. UOM 3 + barley plants;
6. Oil-contaminated soil + *P. hunanensis* IB C7;
7. Oil-contaminated soil + *P. hunanensis* IB C7 + barley plants.

The inoculation of seeds with a liquid culture of bacteria in the amount of 10^6^ CFU per seed (CFU: colony-forming units) took place immediately before sowing. Unbacterized seeds were moistened with water. After treatment, the seeds were planted either in clean or contaminated soil (600 pcs m^−2^) to a depth of 4–5 cm. After that, the plots of variants 4 to 7 were immediately watered with 250 mL of a liquid culture of bacteria (titer 2 × 10^9^ CFU/mL) diluted in 5 L of water. The laboratory seed germination rate was 92%. 

Ten and thirty-four days after the emergence of seedlings, the growth characteristics of the plants were measured. The leaf surface index was assessed by analyzing photographs using ImageJ software [70]. 

At the end of the experiment, the analysis of individual elements of the crop structure was carried out. Since plants were considered only as agents of bioremediation, the use of plant products as food for humans and animals was not envisaged. In accordance, the qualitative and quantitative indicators of grain were not measured.

### 4.2. Analysis of the Content of Pigments and Proline

The content of chlorophyll (a + b) and flavonoids in the leaves was measured using a DUALEX SCIENTIFIC+ device (FORCE-A, Paris, France) according to the manufacturer’s recommendations, and free proline—according to the Bates method [71], using toluene as an extractant. All chemicals used in the work, except those mentioned below, were provided by Merck (Darmstadt, Germany).

### 4.3. Cultivation of the Microorganisms and Analysis of their Number

Bacteria *Enterobacter* sp. UOM 3 and *P. hunanensis* IB C7 were cultured for 72 h in a meat-peptone broth (g/L): peptone–5, NaCl–5 [72] (manufacturer Merck, Germany), at a temperature of 28 °C. Aeration of the medium was provided by rotating flasks (160 rpm) in an orbital shaker-incubator ES-20/60 (SIA BIOSAN, Riga, Latvia). The number of cells in the culture was measured by applying serial dilutions to the nutrient agar (g/L): peptone–10, yeast extract–3, NaCl–5, glucose–1, agar-agar–15 [72] (manufacturer Merck, Germany) and then counting the number of colony-forming units (CFU).

In order to estimate the microbial counts in soil, a serial dilution of soil suspension was used. The number of heterotrophic microorganisms was measured by application to the nutrient agar (see above). For measuring the number of petroleum degrading bacteria, we used Raymond agar (g/L): NH_4_NO_3_–2.0, MgSO_4_ × 7H_2_O–0.2, KH_2_PO_4_–2.0, Na_2_HPO_4_–3, CaCl_2_ × 6H_2_O–0.01, Na_2_CO_3_–0.1, agar-agar–15, pH–7.0) [73], supplemented with 0.1 g of sterile diesel fuel as the only source of carbon, smeared on the agar surface of each plate. To measure the number of oligonitrophilic microorganisms, we used Ashby medium (g/L): mannitol–20, K_2_HPO_4_–0.2, MgSO_4_ × 7H_2_O–0.2, NaCl–0.2, K_2_SO_4_–0.1, CaCO_3_–5, agar-agar–15 [72] (manufacturer Sisco Research Laboratories, India). The incubation period at 28 °C was three days on nutrient agar, five days on the Raymond agar plate, and five days on the Ashby agar. The average number of colonies was calculated in ten agar plates. 

### 4.4. Analysis of the Content of Hydrocarbons in the Soil

Total petroleum hydrocarbons in the soil samples were measured using the EPA 3540C method. Then, 10 g of soil samples were packed in filter paper and extracted in a Soxhlet extractor with 300 mL of hexane for 8 h at six extraction cycles per hour. The extraction product was transferred to a glass column filled with glass wool and Na_2_SO_4_ to remove any water it contained. The extract was collected in a flask for subsequent evaporation of the solvent using a rotary evaporator Rotavapor R-100 (Buchi Labortechnik AG, Flawil, Switzerland) until a final volume of 2 mL was reached. The concentrated solution was poured into a pre-weighed glass beaker and dried until a constant weight was reached. The total petroleum hydrocarbons present in the samples were then quantified by gravimetric analysis with a weighing accuracy of up to 0.1 mg.

### 4.5. Hormone Measurement

Ten days after the emergence of shoots, the concentration of hormones in the shoots and roots was assessed. IAA and abscisic acid (ABA) were extracted according to [74,75]. Purification and analysis of cytokinins (zeatin, its riboside, and nucleotide) were performed according to [76]. The hormone content was determined by ELISA using the appropriate antibodies [37]. 

### 4.6. Statistical Analysis

The data were processed using Statistica (Statsoft) software (version 10). In figures and tables, data are presented as mean ± standard error. The significance of differences was assessed by ANOVA followed by Duncan’s test (*p* ≤ 0.05). 

## 5. Conclusions

During the field experiment, it was shown that, against the background of oil pollution, the simultaneous use of auxin-producing bacterial oil destructors *P. hunanensis* IB C7 and *Enterobacter* sp. UOM 3 and barley plants contributed to a more significant reduction in the content of hydrocarbons in the soil compared to the use of bacteria and plants separately. A good survival rate of introduced bacteria in oil-contaminated soil and a positive effect of bacterization on the growth of barley plants have been established. Treatment with microorganisms mitigated the negative effects of abiotic stress caused by the presence of oil in the soil for plants due to the influence exerted on the hormonal status of plants, as well as on the systems of osmoregulation and photosynthesis.

## Figures and Tables

**Table 1:** The mass of shoots and roots of barley plants 10 days after germination, mg.

Variants of Experiments	Fresh Mass		Root Mass/ Shoot Mass
	Root	Shoot	
Control	34.4 ± 2.0 ^b^	318.4 ± 7.5 ^c^	0.11 ± 0.012 ^a^
Oil	25.1 ± 1.1 ^a^	64.9 ± 1.0 ^a^	0.39 ± 0.080 ^b^
Oil + UOM 3	30.1 ± 2.1 ^ab^	80.3 ± 4.3 ^ab^	0.37 ± 0.058 ^b^
Oil + IB C7	41.9 ± 0.8 ^c^	85.9 ± 2.8 ^b^	0.49 ± 0.043 ^b^

Statistically different means values for each indicator (n = 15) are marked with different letters (*p* ≤ 0.05). UOM 3 and IB C7, variants of experiments with introduction of *Enterobacter* sp. UOM 3 and *P. hunanensis* IB C7, respectively.

**Table 2:** The influence of oil pollution and treatment with bacteria on some indicators of growth and development of barley plants at the end of the experiment.

Variants of Experiments	Bushiness (pcs)	Shoot Height (cm)	The Number of Ears (pcs)	The Length of the Main Spike (cm)	The Number of Spikelets per Spike (pcs)	Dry Mass of the Shoot (g)
Control	4.96 ± 0.28 ^b^	52.24 ± 1.34 ^c^	2.96 ± 0.26 ^c^	6.50 ± 0.23 ^d^	15.82 ± 0.65 ^c^	0.496 ± 0.022 ^c^
Oil	1.80 ± 0.05 ^a^	28.00 ± 0.45 ^a^	1.32 ± 0.05 ^a^	1.87 ± 0.08 ^a^	5.38 ± 0.20 ^a^	0.185 ± 0.009 ^a^
Oil + UOM 3	1.93 ± 0.06 ^a^	29.44 ± 0.47 ^b^	1.43 ± 0.05 ^a^	2.57 ± 0.07 ^c^	6.06 ± 0.19 ^b^	0.213 ± 0.009 ^b^
Oil + IB C7	1.91 ± 0.06 ^a^	28.82 ± 0.41 ^ab^	1.61 ± 0.05 ^b^	2.25 ± 0.07 ^b^	5.69 ± 0.20 ^ab^	0.213 ± 0.010 ^b^

Statistically different means values for each indicator (n = 200) are marked with different letters (*p* ≤ 0.05). UOM 3 and IB C7, variants of experiments with introduction of *Enterobacter* sp. UOM 3 and *P. hunanensis* IB C7, respectively.

**Table 3:** The number of microorganisms in the oil-contaminated soil, CFU/g.

Variants of Experiments		Heterotrophic Microorganisms, ×10^7^		Hydrocarbon-Oxidizing Microorganisms, ×10^6^		Oligonitrophilic Microorganisms, ×10^5^	
		34 Days after Germination	94 Days after Germination	34 Days after Germination	94 Days after Germination	34 Days after Germination	94 Days after Germination
Without plants	Without bacteria	(1.1 ± 0.2) ^a^	(1.2 ± 0.2) ^a^	(1.4 ± 0.3) ^a^	(1.5 ± 0.3) ^a^	(0.3 ± 0.1) ^a^	(2.0 ± 0.1) ^a^
	UOM 3	(1.6 ± 0.4) ^ab^	(2.4 ± 0.6) ^bc^	(7.2 ± 1.5) ^c^	(13.8 ± 3.9) ^c^	(2.0 ± 0.3) ^b^	(9.1 ± 1.2) ^b^
	IB C7	(1.6 ± 0.2) ^ab^	(3.0 ± 0.6) ^bc^	(6.7 ± 2.0) ^c^	(14.3 ± 3.0) ^c^	(1.8 ± 0.3) ^b^	(10.6 ± 1.5) ^b^
With plants	Without bacteria	(1.8 ± 0.1) ^b^	(2.1 ± 0.3) ^b^	(2.7 ± 0.2) ^b^	(3.3 ± 0.4) ^b^	(1.9 ± 0.2) ^b^	(8.5 ± 0.6) ^b^
	UOM 3	(2.3 ± 0.4) ^bc^	(4.6 ± 0.4) ^d^	(19.3 ± 2.9) ^d^	(25.5 ± 2.9) ^d^	(3.9 ± 0.5) ^c^	(29.9 ± 2.3) ^d^
	IB C7	(3.0 ± 0.3) ^c^	(3.3 ± 0.4) ^c^	(21.9 ± 2.4) ^d^	(29.5 ± 3.8) ^d^	(4.1 ± 0.4) ^c^	(20.8 ± 2.4) ^c^

Statistically different means values for each indicator are marked with different letters (*p* ≤ 0.05). UOM 3 and IB C7, variants of experiments with introduction of *Enterobacter* sp. UOM 3 and *P. hunanensis* IB C7, respectively.

**Table 4:** Biochemical indicators of barley plants.

Variants of Experiments	Chlorophyll (μg/cm^2^)	Flavonoids (a.u.)	NBI (a.u.)	Proline (μg/g)
10 days after germination				
Control	35.1 ± 1.0 ^e^	0.56 ± 0.02 ^a^	62.7 ± 2.0 ^f^	26.4 ± 3.3 ^a^
Oil	16.3 ± 1.0 ^a^	0.90 ± 0.02 ^e^	18.1 ± 1.0 ^a^	79.3 ± 2.8 ^c^
Oil + UOM 3	18.4 ± 1.0 ^ab^	0.89 ± 0.02 ^e^	20.7 ± 1.0 ^b^	29.5 ± 2.9 ^a^
Oil + IB C7	18.2 ± 1.0 ^ab^	0.87 ± 0.02 ^e^	20.9 ± 1.0 ^b^	35.5 ± 3.2 ^a^
34 days after germination				
Control	32.0 ± 0.6 ^d^	0.65 ± 0.02 ^b^	49.2 ± 1.4 ^e^	52.9 ± 4.5 ^b^
Oil	18.9 ± 0.7 ^b^	0.74 ± 0.01 ^c^	24.7 ± 1.0 ^c^	115.9 ± 4.2 ^d^
Oil + UOM 3	22.1 ± 0.9 ^c^	0.81 ± 0.01 ^d^	27.3 ± 0.8 ^c^	61.9 ± 2.7 ^b^
Oil + IB C7	24.1 ± 0.5 ^c^	0.79 ± 0.01 ^d^	30.5 ± 0.6 ^d^	80.7 ± 3.4 ^c^

Statistically different means values for each indicator are marked with different letters (*p* ≤ 0.05). UOM 3 and IB C7, variants of experiments with introduction of *Enterobacter* sp. UOM 3 and *P. hunanensis* IB C7, respectively.

## Data Availability

The data presented in this study are available in the graphs and tables provided in the manuscript.
